# Supplementary material for: Local chromatin fiber folding represses transcription and loop extrusion in quiescent cells
Source: eLife. 2021 Nov 4;10:e72062. doi: 10.7554/eLife.72062 (PMC8598167; doi:10.7554/eLife.72062)

Figure 3-supplement 1 Western blot source data

Relevant lanes are marked with brackets and bands are marked with an arrow.

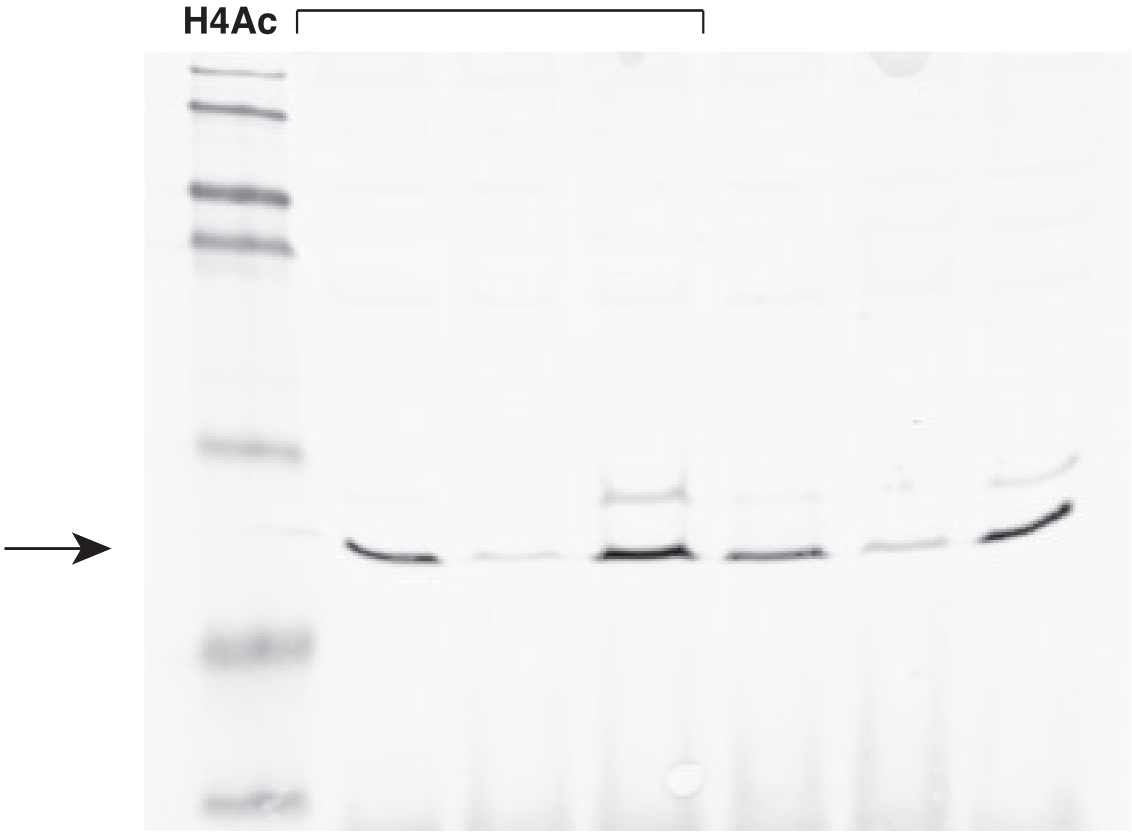

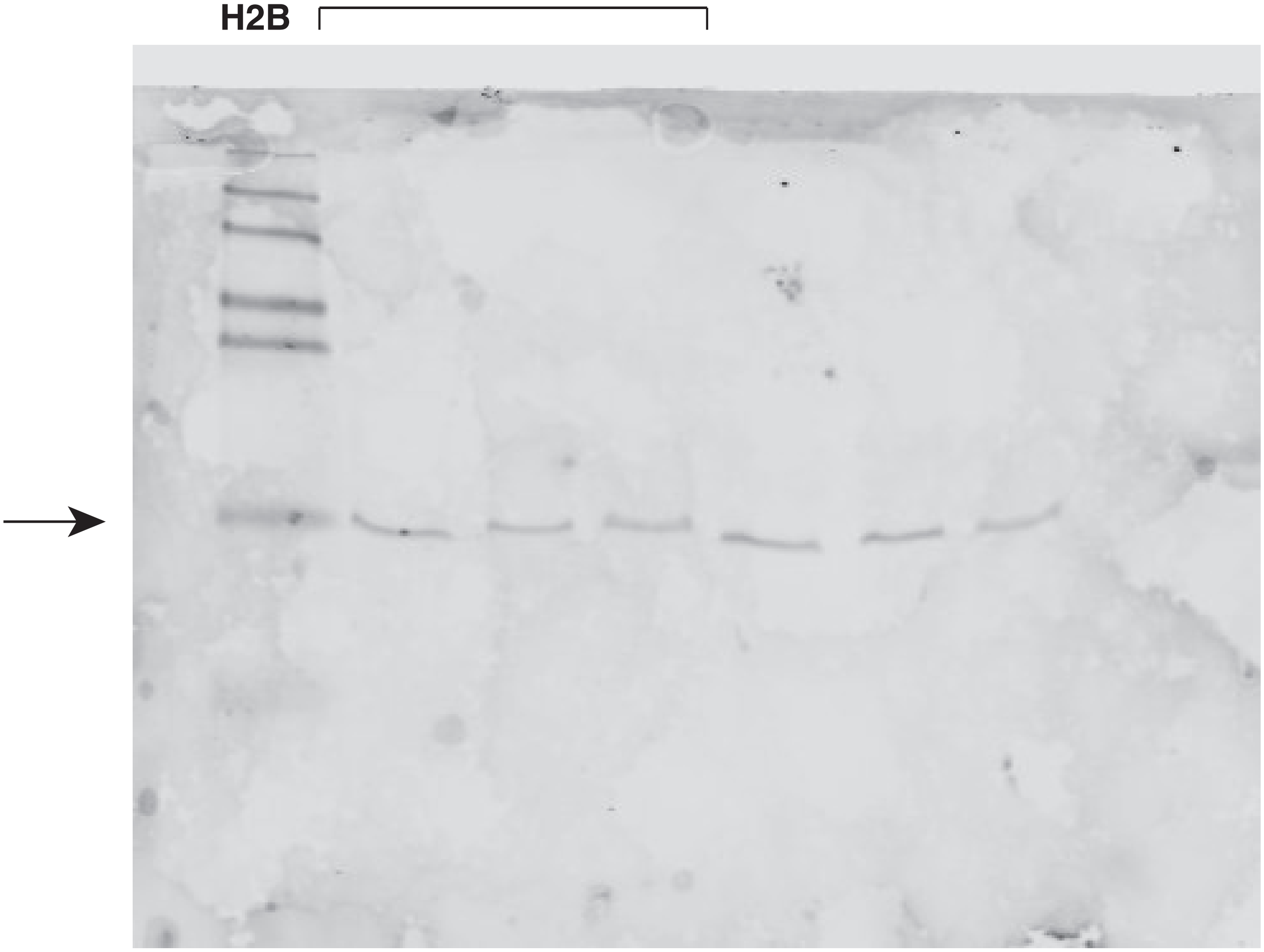

Supplement: Figure 3—figure supplement 1—source data 1. [file elife-72062-fig3-figsupp1-data1.pdf]
