# Supplementary material for: Local chromatin fiber folding represses transcription and loop extrusion in quiescent cells
Source: eLife. 2021 Nov 4;10:e72062. doi: 10.7554/eLife.72062 (PMC8598167; doi:10.7554/eLife.72062)
Supplement: Supplementary file 1. [file elife-72062-supp1.docx]

**Supplementary Table 1**

| **Genotype/**  **Condition** | **Replicate** | | **n** | | **Mean** | **SD** | | **Combined n** | | **Combined Mean** | | **Combined SD** |
| --- | --- | --- | --- | --- | --- | --- | --- | --- | --- | --- | --- | --- |
| WT Q | 5781 | | 266 | | 0.6866 | 0.1525 | | 381 | | 0.6852 | | 0.1468 |
|  | 5783 | | 115 | | 0.6822 | 0.133 | |  |  |  |  |  |
| WT + TSA Q | 5781 + TSA | | 241 | | 0.8463 | 0.1831 | | 923 | | 0.8468 | | 0.2047 |
|  | 5783 + TSA | | 682 | | 0.8469 | 0.2119 | |  |  |  |  |  |
| HHF2 Q | 7177 | | 375 | | 0.6775 | 0.119 | | 585 | | 0.6844 | | 0.1331 |
|  | 7206 | | 210 | | 0.6967 | 0.1548 | |  |  |  |  |  |
| K16A Q | 7202 | | 451 | | 0.8374 | 0.1398 | | 861 | | 0.8168 | | 0.126 |
|  | 7209 | | 410 | | 0.7942 | 0.1043 | |  |  |  |  |  |
| K16Q Q | 7205 | | 357 | | 0.8295 | 0.1441 | | 867 | | 0.8188 | | 0.1268 |
|  | 7210 | | 510 | | 0.8113 | 0.1126 | |  |  |  |  |  |
| R17R19A Q | 7200 | | 394 | | 0.914 | 0.161 | | 765 | | 0.9139 | | 0.15 |
|  | 7207 | | 371 | | 0.9139 | 0.1375 | |  |  |  |  |  |
| 5toA Q | 7175 | | 384 | | 0.9702 | 0.1715 | | 749 | | 0.9662 | | 0.1707 |
|  | 7208 | | 365 | | 0.962 | 0.17 | |  |  |  | |  |
| HTA1 Q | 6767 | | 294 | | 1.078 | 0.2775 | | 422 | | 1.081 | | 0.2708 |
|  | 6773 | | 128 | | 1.087 | 0.2558 | |  |  |  | |  |
| E65D92A Q | 6618 | | 441 | | 1.393 | 0.3032 | | 591 | | 1.387 | | 0.3054 |
|  | 6765 | | 150 | | 1.37 | 0.3122 | |  |  |  | |  |
| E57A Q | 6768 | | 165 | | 1.394 | 0.3422 | | 258 | | 1.393 | | 0.3433 |
|  | 6774 | | 93 | | 1.391 | 0.3471 | |  |  |  | |  |
| E65A Q | 6769 | | 210 | | 1.292 | 0.2752 | | 420 | | 1.305 | | 0.2784 |
|  | 6776 | | 210 | | 1.319 | 0.2816 | |  |  |  | |  |
| E93A Q | 6772 | | 147 | | 1.294 | 0.3218 | | 410 | | 1.307 | | 0.339 |
|  | 6779 | | 263 | | 1.315 | 0.3486 | |  |  |  | |  |
| WT G1 | 5781 G1 | | 182 | | 1.092 | 0.2811 | | 621 | | 1.093 | | 0.2665 |
|  | 5783 G1 | | 439 | | 1.094 | 0.2605 | |  |  |  | |  |
| R17R19A G1 | 7200 G1 | | 134 | | 1.078 | 0.3348 | | 280 | | 1.06 | | 0.3558 |
|  | 7207 G1 | | 146 | | 1.043 | 0.3745 | |  |  |  | |  |
| 5toA G1 | 7175 G1 | | 110 | | 1.077 | 0.3704 | | 287 | | 1.06 | | 0.39 |
|  | 7208 G1 | | 177 | | 1.05 | 0.4025 | |  |  |  | |  |
| 5toA Q + Phe | 7175 + Phe | | 213 | | 0.9642 | 0.1652 | | 377 | | 0.9591 | | 0.192 |
|  | 7208 + Phe | | 164 | | 0.9525 | 0.2225 | |  |  |  | |  |
| **Games-Howell's multiple comparisons test** | | **Mean Diff.** | | **95.00% CI of diff.** | | | **t** | | **DF** | | **Adjusted P Value** | |
| G1 vs. Q | | 0.4079 | | 0.3772 to 0.4386 | | | 31.2 | | 989.9 | | <0.0001 | |
| G1 vs. Q + TSA | | 0.2464 | | 0.2167 to 0.2760 | | | 19.49 | | 1094 | | <0.0001 | |
| Q vs. Q + TSA | | -0.1615 | | -0.1852 to  -0.1378 | | | 16 | | 975.9 | | <0.0001 | |
| HHF2 vs. K16A | | -0.1324 | | -0.1515 to  -0.1134 | | | 18.97 | | 1207 | | <0.0001 | |
| HHF2 vs. K16Q | | -0.1344 | | -0.1535 to  -0.1153 | | | 19.24 | | 1212 | | <0.0001 | |
| HHF2 vs. R17,R19A | | -0.2296 | | -0.2507 to  -0.2084 | | | 29.71 | | 1318 | | <0.0001 | |
| HHF2 vs. 5toA | | -0.2818 | | -0.3046 to  -0.2591 | | | 33.88 | | 1332 | | <0.0001 | |
| K16A vs. K16Q | | -0.001992 | | -0.01860 to 0.01461 | | | 0.3276 | | 1726 | | 0.9975 | |
| K16Q vs. R17,R19A | | -0.09512 | | -0.1140 to  -0.07621 | | | 13.74 | | 1504 | | <0.0001 | |
| R17,R19A vs. 5toA | | -0.0523 | | -0.07487 to  -0.02972 | | | 6.328 | | 1479 | | <0.0001 | |
| WT Q vs. WT G1 | | -0.4079 | | -0.4452 to  -0.3706 | | | 31.2 | | 989.9 | | <0.0001 | |
| R17,R19A Q vs. R17,19A G1 | | -0.1458 | | -0.2088 to  -0.08292 | | | 6.646 | | 316 | | <0.0001 | |
| 5toA Q vs. 5toA G1 | | -0.09392 | | -0.1623 to  -0.02554 | | | 3.937 | | 328.8 | | 0.0014 | |
| WT G1 vs. R17,19A G1 | | 0.03337 | | -0.03477 to 0.1015 | | | 1.402 | | 425.7 | | 0.7259 | |
| WT G1 vs. 5toA G1 | | 0.033 | | -0.03968 to 0.1057 | | | 1.3 | | 413.8 | | 0.785 | |
| R17,19A G1 vs. 5toA G1 | | -0.0003688 | | -0.08999 to 0.08926 | | | 0.01177 | | 562.5 | | >0.9999 | |
| HTA1 vs. E65A | | -0.2285 | | -0.2779 to  -0.1790 | | | 12.63 | | 871.7 | | <0.0001 | |
| HTA1 vs. E93A | | -0.2304 | | -0.2866 to  -0.1742 | | | 11.2 | | 765.7 | | <0.0001 | |
| HTA1 vs. E57A | | -0.3164 | | -0.3835 to  -0.2493 | | | 12.92 | | 421.1 | | <0.0001 | |
| HTA1 vs. E65,D92A | | -0.3102 | | -0.3576 to  -0.2629 | | | 17.9 | | 1078 | | <0.0001 | |
| E65A vs. E93A | | -0.001958 | | -0.06090 to 0.05699 | | | 0.09081 | | 790.4 | | >0.9999 | |
| E93A vs. E57A | | -0.08598 | | -0.1603 to  -0.01168 | | | 3.167 | | 541.1 | | 0.014 | |
| E57A vs. E65,D92A | | 0.006177 | | -0.06173 to 0.07408 | | | 0.2492 | | 442.3 | | 0.9991 | |
| **Welch's t test** | | **Mean Diff.** | | **95.00% CI of diff.** | | | **Welch-corrected t** | | **DF** | | **P Value** | |
| 5toA + Phe vs 5toA | | -0.0071 | | -0.03010 to 0.01582 | | | 0.6108 | | 680.4 | | 0.5415 | |
